# Estimating energetics in cetaceans from respiratory frequency: why we need to understand physiology

**DOI:** 10.1242/bio.017251

**Published:** 2016-03-17

**Authors:** A. Fahlman, J. van der Hoop, M. J. Moore, G. Levine, J. Rocho-Levine, M. Brodsky

**Affiliations:** 1 Texas A&M University – Corpus Christi, 6300 Ocean Drive, Corpus Christi, TX 78412, USA; 2 Massachusetts Institute of Technology – Woods Hole Oceanographic Institution Joint Program in Oceanography, 77 Massachusetts Ave, Cambridge, MA 02139, USA; 3 Biology Department, Woods Hole Oceanographic Institution, 266 Woods Hole Rd, Woods Hole, MA 02543, USA; 4 Dolphin Quest, Oahu, 5000 Kahala Ave, Honolulu, HI 96816, USA; 5 V.M.D. Consulting, Miami, FL 33138, USA; 6 Oceanográfic, Research Department , Carrer Eduardo Primo Yúfera 1B, Valencia 46012, Spain

**Keywords:** Field metabolic rate, Marine mammal, Oxygen consumption rate, Exercise, Recovery, Eco-physiology, Energy budget, Oxygen debt

## Abstract

The accurate estimation of field metabolic rates (FMR) in wild animals is a key component of bioenergetic models, and is important for understanding the routine limitations for survival as well as individual responses to disturbances or environmental changes. Several methods have been used to estimate FMR, including accelerometer-derived activity budgets, isotope dilution techniques, and proxies from heart rate. Counting the number of breaths is another method used to assess FMR in cetaceans, which is attractive in its simplicity and the ability to measure respiration frequency from visual cues or data loggers. This method hinges on the assumption that over time a constant tidal volume (VT) and O_2_ exchange fraction (ΔO_2_) can be used to predict FMR. To test whether this method of estimating FMR is valid, we measured breath-by-breath tidal volumes and expired O_2_ levels of bottlenose dolphins, and computed the O_2_ consumption rate (*V̇*_O_2__) before and after a pre-determined duration of exercise. The measured *V̇*_O_2__ was compared with three methods to estimate FMR. Each method to estimate *V̇*_O_2__ included variable VT and/or ΔO_2_. Two assumption-based methods overestimated *V̇*_O_2__ by 216-501%. Once the temporal changes in cardio-respiratory physiology, such as variation in VT and ΔO_2_, were taken into account, pre-exercise resting *V̇*_O_2__ was predicted to within 2%, and post-exercise *V̇*_O_2__ was overestimated by 12%. Our data show that a better understanding of cardiorespiratory physiology significantly improves the ability to estimate metabolic rate from respiratory frequency, and further emphasizes the importance of eco-physiology for conservation management efforts.

## Abstract (precis)

**Summary:** Accounting for changes in tidal volume and gas exchange improves the ability to estimate field metabolic rate from respiratory frequency in cetaceans.

## INTRODUCTION

Marine mammals live a life of dual constraints, with food located underwater, and the oxygen (O_2_) required to fuel aerobic metabolism available only at the surface. Empirical data and optimal foraging theory suggests that aerobic dives should maximize time underwater, thereby increasing foraging efficiency (Carbone and Houston, 1996; Kooyman et al., 1983). For an animal that mainly utilizes aerobic metabolism, the time available to forage underwater depends on the amount of O_2_ available in the lung, blood and tissue stores, and the rate at which these stores are utilized (the metabolic rate).

Marine mammals have several traits that enhance time underwater such as: increased muscle and blood O_2_ stores, a shape that minimizes hydrodynamic drag, and a physiological response during diving that helps to manage gases and maximize the aerobic dive time (Butler and Jones, 1997; Ponganis et al., 2011; Scholander, 1940). While many of these traits have been described for a range of pinnipeds and smaller cetaceans (Fahlman et al., 2008a; Kooyman et al., 1971; Reed et al., 1994, 2000; Sparling and Fedak, 2004; Sparling et al., 2007; Williams et al., 1993; Yazdi et al., 1999), we still know very little how larger, free ranging cetaceans manage their energy budgets (Kooyman et al., 1975; Sumich, 2001; Wahrenbrock et al., 1974).

Energy budgets of free-ranging animals are critical variables in bioenergetics models used to assess important issues such as the impact of climate change, or variation in food distribution, on marine mammal populations (Bowen, 1997; Winship et al., 2002). Current bioenergetics models are more sensitive to uncertainty in energy budgets and metabolic rates than to diet composition or population size (Winship et al., 2002). Better resolution of energy budgets requires an understanding of how animals manage their time underwater, their physiological limitations, and the energy expenditures associated with different activities.

Estimating the field metabolic rate (FMR) in breath-hold diving animals is challenging, as it is logistically difficult to investigate free-ranging animals, especially when they spend considerable time underwater and migrate over large areas. Several indirect methods have been used to assess FMR in diving species (Dolphin, 1987a; Fahlman et al., 2013; Folkow and Blix, 1992; Hindell and Lea, 1998). These include the use of stable isotopes (Boyd et al., 1995; Costa, 1988), and relating activity levels or heart rate to metabolic rates in calibrated studies (Boyd et al., 1995; Butler et al., 2004; Fahlman et al., 2013, 2008b; Halsey et al., 2008; McPhee et al., 2003; Williams et al., 2004; Young et al., 2011a,b).

Another approach that has been used is to assume that respiratory frequency should relate to minute volume or energetic demand (Armstrong and Siegfried, 1991; Blix and Folkow, 1995; Christiansen et al., 2014; Dolphin, 1987a,b; Folkow and Blix, 1992; Williams and Noren, 2009). FMR of humpback whales (*Megaptera novaeangliae*, Dolphin, 1987a), minke whales, (*Balaenoptera acutorostrata*, Armstrong and Siegfried, 1991; Blix and Folkow, 1995; Christiansen et al., 2014; Folkow and Blix, 1992), gray whales (*Eschrichtius robustus*, Sumich, 1983), and killer whales (*Orcinus orca*, Williams and Noren, 2009) have been estimated by counting the number of breaths during a surface interval. These methods use a constant value for the tidal volume (VT) and O_2_ exchange fraction (ΔO_2_=inspired O_2_−expired O_2_). While it is known that both vary with activity level and following diving (Fahlman et al., 2015; Reed et al., 1994, 2000), it may be valid to assume that over time, average values of these parameters can be used to estimate FMR from breathing frequency. This method to estimate FMR is attractive in its simplicity, but the assumptions about VT and ΔO_2_ have not been validated. While validation experiments are logistically difficult in larger cetaceans, the current study sought to determine how accurate we could estimate metabolic cost during rest and following a bout of aerobic exercise. The vital capacity (VC) of cetaceans is high and estimated to be approximately 80-90% of their total lung capacity (TLC) (Fahlman et al., 2015; Kooyman and Cornell, 1981; Kooyman et al., 1975; Olsen et al., 1969). Some studies have assumed that VT is independent of activity level and is close to VC (Dolphin, 1987a; Williams and Noren, 2009). If all breaths are close to VC, increased O_2_ demand, as occurs during periods of increased activity, would be achieved through an increase in breathing frequency (Kooyman et al., 1971). Based on these relationships, it has been expected that breathing frequency is tightly linked to energy requirements and may be used to assess or compare the metabolic cost of different activities, e.g. resting or swimming at the surface versus diving.

**List of abbreviations (units)**

AIC: Akaike information criterion
BN: Breath number
ΔO_2_: Oxygen extraction (inspired oxygen fraction – expired oxygen fraction, %)
P_O_2__: Partial pressure of O_2_ (kPa)
s *V̇*_O_2__: Mass-specific oxygen consumption rate (ml O_2_ min^−1^ kg^−1^)
STPD: Standard temperature pressure dry (0°C, 100 kPa, 0% humidity)
TLC: Total lung capacity (litre)
VC: Vital capacity (litre)
VT: Tidal volume (litre)
*V̇*_CO_2__: Carbon dioxide production rate (litre CO_2_ min^−1^)
*V̇*_O_2__: Oxygen consumption rate (litre O_2_ min^−1^)

However, there appears to be considerable variability in VT during spontaneous breaths in both small (Fahlman et al., 2015; Olsen et al., 1969; Sumich, 2001) and large cetaceans (Wahrenbrock et al., 1974). Tidal volumes of resting pilot whales (*Globicephala scammoni/macrorhynchus*) and bottlenose dolphins (*Tursiops truncatus*) range from 20-88% and 24-40% of TLC, respectively (Fahlman et al., 2015; Olsen et al., 1969). In two publications on gray whales VT was reported to vary by up to 50% between breaths (Wahrenbrock et al., 1974) or to range by two orders of magnitude (1 litre to approximately 180 litre, Sumich, 2001). Like VT, ΔO_2_ is highly variable between breaths, increasing with the preceding breath-hold duration (Reed et al., 2000; Sumich, 2001) and decreasing with breath number following diving (Ridgway et al., 1969). Thus, VT and ΔO_2_ appear to vary both within and between species, which raises concern over the validity of estimating energy expenditure based on static estimates of these parameters.

In order to refine computation of free-swimming marine mammal energetics, we determined how accurately we could estimate FMR in a small cetacean, the bottlenose dolphin, before and after exercise, from respiratory frequency alone. We compared 3 different methods to estimate metabolic rate from breathing frequency and compared the results to experimentally derived measurements. Our results indicate that with an understanding of the underlying physiology, the metabolic rate before and after exercise can be estimated to within 2% and 12%, respectively.

## RESULTS

### Measured respiratory values and rates

Average maximum expiratory flow rates increased after exercise (AICc=512.7, *P*<0.01, PRE=25.5±7.3 litre s^−1^, POST=30.9±7.5 litre s^−1^) but the inspiratory flow rates (AICc=451.9, *P*>0.1, PRE=18.1±3.9 litre s^−1^, POST=19.5±3.8 litre s^−1^) did not differ between pre- and post-exercise samples. Neither inspiratory (AICc=274.1, *P*>0.1, PRE=0.38±0.06 s, POST=0.39±0.06 s), expiratory (AICc=260.5, *P*>0.05, PRE=0.36±0.06 s, POST=0.33±0.08 s), or total breath durations (AICc=170.8, *P*>0.1, PRE=0.75±0.11 s, POST=0.74±0.12 s) changed following exercise. The inter-breath interval (inverse of breathing frequency) was significantly shorter following exercise (AICc=580.6, *P*<0.01, PRE=24.8±8.8 s, POST=17.5±7.6 s). The average VT (AICc=479.7, *P*<0.05) increased significantly following exercise (Table 1).

**Table 1.**
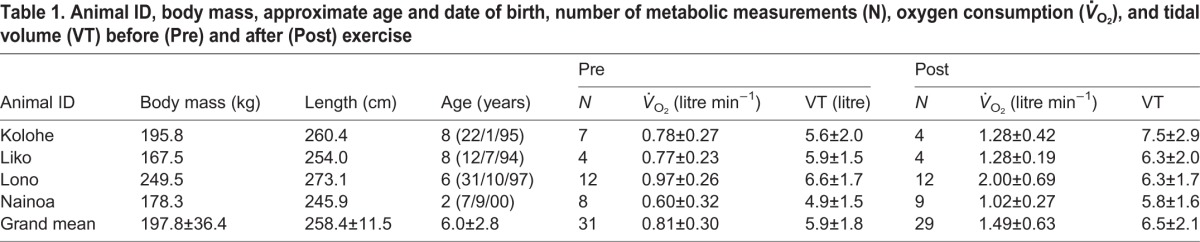
**Animal ID, body mass, approximate age and date of birth, number of metabolic measurements (N), oxygen consumption (*V̇*_O_2__), and tidal volume (VT) before (Pre) and after (Post) exercise**

**Fig. 1.**
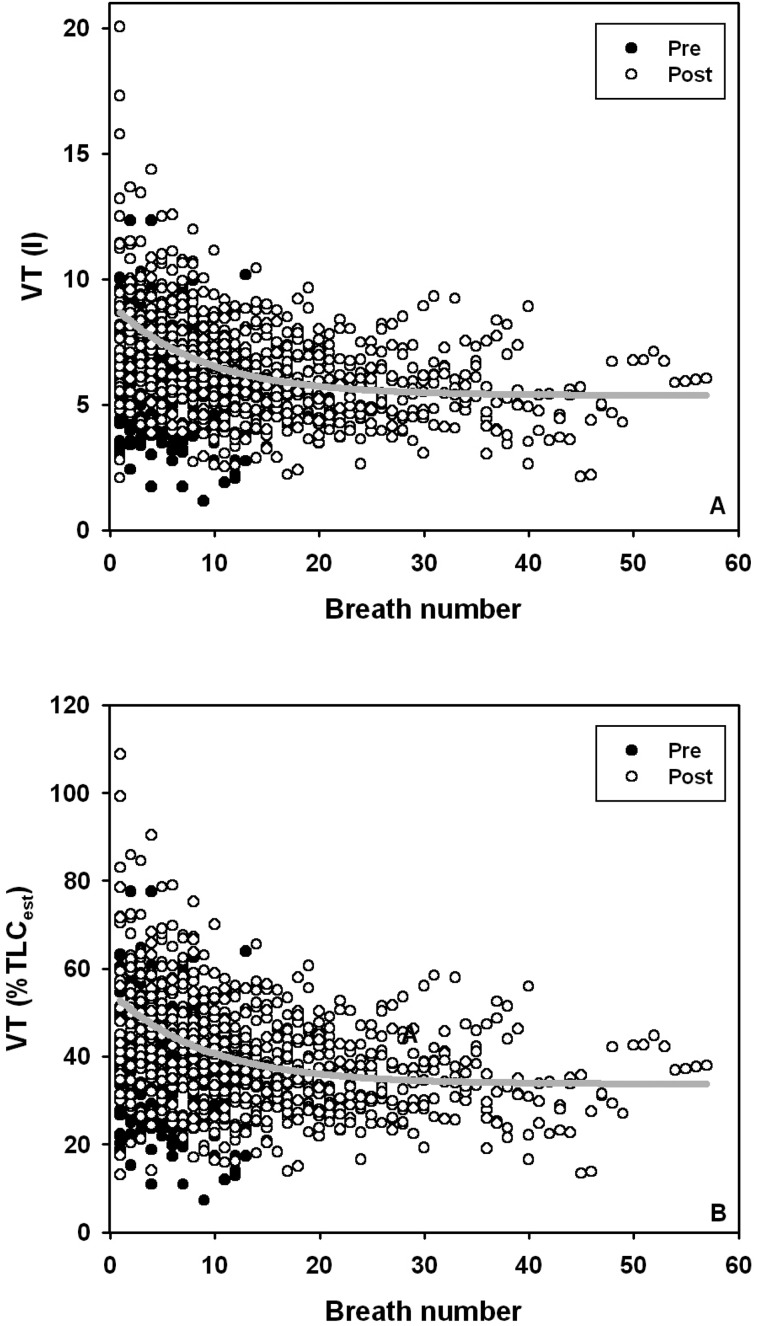
**Changes in tidal volume with breath number**. (A) Absolute tidal volume (VT, litres), or (B) tidal volume expressed as percentage total lung capacity (%TLC_est_) decrease with breath number during the post-exercise recovery period in bottlenose dolphins. Grey line shows exponential relationship (Eqn 1A or Eqn 1B). %TLC_est_=VT·TLC_est_^−1^·100, where TLC_est_=0.135·*M*_b_^0.92^ (Fahlman et al., 2011; Kooyman, 1973).

**Fig. 2.**
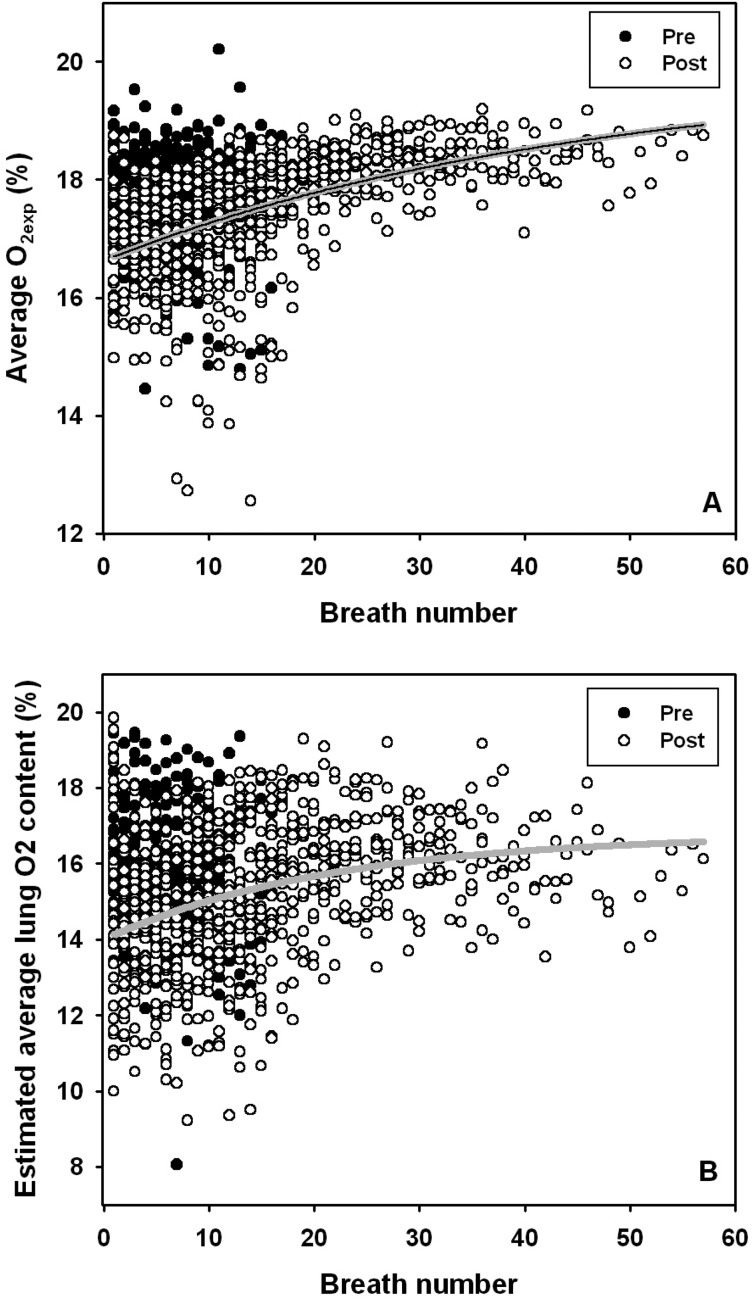
**Changes in lung O_2_ content with breath number.** (A) Average expired O_2_ content (O_2exp_, %) and (B) average estimated lung O_2_ concentration (%) upon expiration, before (Pre) and immediately following (Post) exercise. Data are pooled for all dolphins. Grey line shows exponential relationship (Eqn 1C or Eqn 1D).

During the pre-exercise resting trials, neither the number of breaths, the end-expiratory O_2_, the volume of O_2_ exchanged per breath, average O_2_ content per breath, nor VT changed (mixed model ANOVA, *P*>0.1). Following exercise, VT (Fig. 1), and the volume of O_2_ exchanged per breath decreased, while end-expiratory and average O_2_ content increased (Fig. 2, *P*<0.05).

The average end-expiratory O_2_, and average expired O_2_ content (AICc=254.0, *P*<0.01), were significantly higher before compared with following exercise. During the post exercise recovery period, the VT decreased with breath number, while average O_2_ content, and end-expired O_2_ increased with breath number until reaching a plateau (Figs 1,2). An exponential equation described how the average VT (Fig. 1A; Eqn 1A, *F*_776,2_=104.2, *P*<0.01, r^2^=0.212) changed with breath number (BN) following exercise (the s.e. of the parameter estimate is in parenthesis):1A
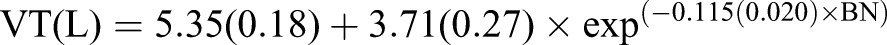


The VT expressed as a percentage of the estimated total lung capacity (Fig. 1B, TLC_est_=0.135×*M*_b_^0.92^; Fahlman et al., 2011; Kooyman, 1973) did not differ before (PRE=32±10% of TLC_est_) or after exercise (POST=34±12%), but decreased with breath number following exercise. The exponential equation that described changes in VT expressed as a percentage of TLC_est_ (%TLC_est_, *F*_776,2_=84.4, *P*<0.01, r^2^=0.174) was:1B
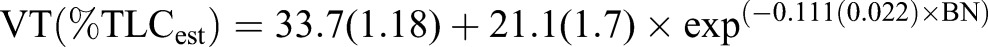
Average O_2exp_ (Fig. 2A; Eqn 1C, *F*_767,2_=148.3, *P*<0.01, r^2^=0.279) also changed with breath number (BN) following exercise (the s.e. of the parameter estimate is in parenthesis):1C
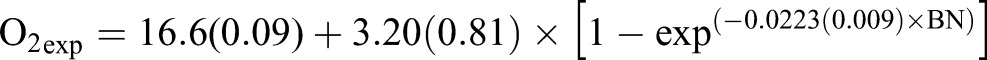
When we used the estimated VT (Eqn 1B) and measured *V̇*_O_2__ for each breath to estimate average O_2exp_, average O_2_ content was 16.0%±1.7% (range 4.0% to 19.5%) and 15.2%±1.8% (range 9.2% to 19.8%, Fig. 2B) for pre- and post-exercise breaths, respectively. There were no changes in estimated lung O_2_ content during rest before exercise, but estimated lung O_2_ content increased with breath number following exercise, described by the exponential equation (*F*_767,2_=55.3, *P*<0.01, r^2^=0.123):1D
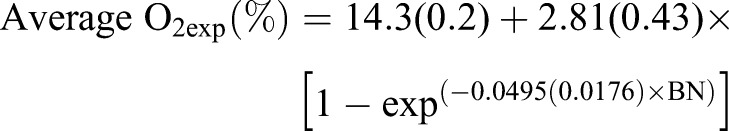
Oxygen consumption rate (*V̇*_O_2__, litre O_2_ min^−1^; AICc=81.2, *P*<0.01, Table 1), mass-specific *V̇*_O_2__ (AICc=316.8, *P*<0.01, PRE=3.96±0.51 ml min^−1^ kg^−1^, POST=6.98±1.05 ml min^−1^ kg^−1^) and 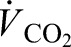 (AICc=302.3, *P*<0.01, PRE=3.48±0.68 ml min^−1^ kg^−1^, POST=5.78±1.24 ml min^−1^ kg^−1^) all increased following exercise.

### Estimated *V̇*_O_2__ from breaths

We used three methods (A-C) to estimate metabolic rate from breathing frequency. Methods A and B used Eqns 2A-C and assumptions used to estimate FMR in free-ranging mysticetes. For method C, we used the measured VT and the integrated volume of O_2_ taken up during each breath to estimate the average O_2_ content for that breath, Eqn 1D. The estimated average O_2exp_ was used to determine average ΔO_2_, which in turn was used to estimate *V*_O_2__ and *V̇*_O_2__ for method C. Thus a mass-independent estimate of respiratory effort (Eqn 1B) was used estimate VT.

#### Pre-exercise rest

Method A and B overestimated the measured *V̇*_O_2__ (0.81 litre min^−1^) by 291% (3.17 litre min^−1^) and 501% (4.88 litre min^−1^), respectively. For method C, the estimated O_2_ content (16.0%) and average VT (32% of TLC_est_) underestimated the measured metabolic rate by 2% (0.79 litre min^−1^).

#### Post-exercise

Following exercise, all methods overestimated the measured *V̇*_O_2__ (1.49 litre min^−1^). Method A and B, using the assumptions described for estimating FMR for humpback whales (Dolphin, 1987a) and minke whales (Armstrong and Siegfried, 1991; Blix and Folkow, 1995; Christiansen et al., 2014; Folkow and Blix, 1992), overestimated *V̇*_O_2__ by 216% (4.72 litre min^−1^) and 382% (7.19 litre min^−1^), respectively. Method C used Eqns 1B and 1D for post-exercise data to estimate *V̇*_O_2__, resulting in an overestimate by 12% (1.67 litre min^−1^). Thus, accounting for post-exercise physiological responses improves the estimated metabolic rate to within 12% during recovery from exercise.

## DISCUSSION

We used breath-by-breath respirometry to determine if counting the number of breaths can be used to estimate metabolic rate in a small cetacean at rest, or during recovery from exercise. Our data clearly indicate that both VT and ΔO_2_ vary following a bout of exercise in bottlenose dolphins. We compared different assumptions to estimate *V̇*_O_2__, most which have been used to estimate FMR in large mysticetes following diving or during migration (Armstrong and Siegfried, 1991; Blix and Folkow, 1995; Christiansen et al., 2014; Dolphin, 1987a,b; Folkow and Blix, 1992). These methods overestimated measured metabolic rates by 216-501%. Accounting for temporal changes following exercise, we created a model with which we were able to estimate the resting and post-exercise recovery metabolic rate to within 2% and 12%, respectively (Method C). While our data suggest that improved knowledge of the physiology of these animals significantly enhances the ability to more accurately predict energy needs, there are some important caveats to consider. First, we studied a small odontocete and both the differences in size (allometry) and physiology warrant consideration. Second, these additional data were performed on dolphins during rest and following a standardized bout of aerobic exercise at the surface. Thus, direct comparisons with diving animals may not be valid.

Similar to our findings, previous data in both pinnipeds (Kerem et al., 1975; Kooyman et al., 1971; Reed et al., 1994) and cetaceans (Reed et al., 2000; Ridgway et al., 1969) indicate that both ΔO_2_ (Fig. 1) and VT (Fig. 2) change significantly as animals recover from exercise. Method A and B, which have been used to estimate FMR in humpback whales (Dolphin, 1987a) and minke whales (Armstrong and Siegfried, 1991; Blix and Folkow, 1995; Christiansen et al., 2014; Dolphin, 1987a,b; Folkow and Blix, 1992), overestimated *V̇*_O_2__ by between 216-501%. These methods assume that all breaths during a surface interval have an average VT between 60-80% of VC and that ΔO_2_ is 10-11.6% (Armstrong and Siegfried, 1991; Blix and Folkow, 1995; Christiansen et al., 2014; Dolphin, 1987a,b; Folkow and Blix, 1992). There are a number of possible differences that may explain this large error such as species or allometric differences, and the difference in behaviors investigated.

Our data from bottlenose dolphins in this and our previous study (Fahlman et al., 2015) show that in this small odontocete most voluntary breaths and maximal VTs following a bout of aerobic exercise range from 25-45% and 63-95% of TLC, respectively, or between 27-102% of VC assuming a minimum air volume of 7% of TLC (Fahlman et al., 2015). Only seven breaths following exercise exceeded 80% of TLC_est_, and most breaths were only about 37-40% of TLC_est_ (Fig. 1B). Cetaceans have the capacity to exchange much of their TLC, and probably do so at times for a few breaths following a long dive or intense exercise, allowing them to rapidly replenish the O_2_ stores and reduce the surface interval and recovery period (Boutilier et al., 2001; Fahlman et al., 2008a). However, we observed considerable variation in VT and average values far below 60-80% of VC (Fig. 1B). Thus, the assumed average VT in Method A and B is a major contributor to its large error and overestimation of metabolic rate. As our experimental design only involved experiments on a small cetacean during rest and recovery from exercise, this conclusion may have to be viewed with caution as there may be considerable differences during versus after exercise and between species, especially large cetaceans.

The VT has been reported to vary considerably in large cetaceans both at rest and following apnea (Epple et al., 2015; Kasting et al., 1989; Kooyman et al., 1975; Sumich and May, 2009; Wahrenbrock et al., 1974; B. Kriete, PhD thesis, The University of British Columbia, 1995). The average VT for a gray whale in human care (Gigi II) was 220 litres when she weighed 6200 kg (Wahrenbrock et al., 1974). At this size, TLC_est_ would be approximately 416 litres, and with a minimum air volume of 7% of TLC, VC would be 387 litres (Fahlman et al., 2011; Kooyman, 1973). Thus, the average VT was about 57% of VC. Similar calculations during growth (2000-6200 kg) showed that average VT increased steadily from 15% to 55% of VC (Wahrenbrock et al., 1974). Similar calculations for the killer whale and beluga whale (*Delphinapterus leucas*) suggest that VT ranges from 35-95% of VC in the former (Kasting et al., 1989; B. Kriete, PhD thesis, The University of British Columbia, 1995) and from 23-80% in the latter (Epple et al., 2015; Kasting et al., 1989). Consequently, this large variation between studies may at least in part explain the large difference between observed *V̇*_O_2__ and that estimated from Methods A and B.

Another reason for the large error for Method B stems from the method to estimate VC (Table 2). For example, the range of estimated VC for Method A ranged from 10.3-15.5 litres for animals ranging from 168-250 kg, while for Method B the range was 19.2-26.6 litres. Thus, the equation for VC in Method B may only be valid for minke whales and inappropriate for smaller cetaceans.

**Table 2.**
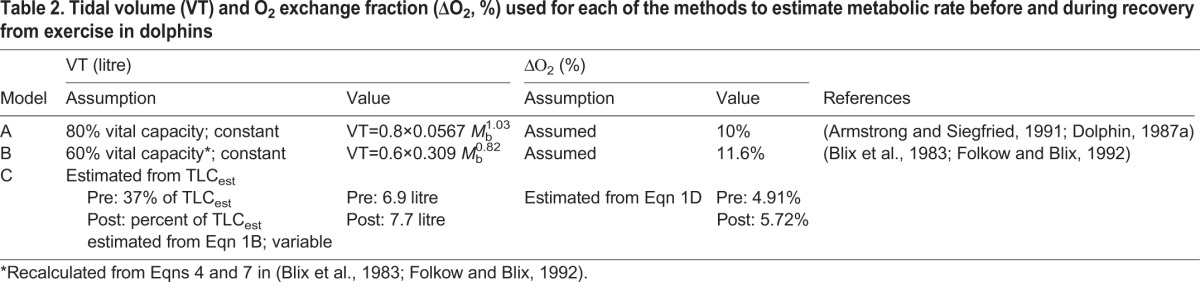
**Tidal volume (VT) and O_2_ exchange fraction (ΔO_2_, %) used for each of the methods to estimate metabolic rate before and during recovery from exercise in dolphins**

Additional error may for Methods A and B may stem from the value assumed average O_2_ content of the lung of 10 and 11.6%, respectively (10 and 11.6 kPa). In previous studies the end-tidal P_O_2__ in the gray whale ranged from 7.1 to 11.2 kPa (Wahrenbrock et al., 1974), and from 7.5 to 16.0 kPa in the harbour porpoise (Reed et al., 2000). In the current study the end-expired O_2_ ranged from 8.1 to 17.8 kPa and from 5.8 to 18.1 kPa before and after exercise, respectively. Thus, our end-expiratory values are within the range of those previously reported for both a small and a large cetacean. In another study, average O_2_ content of 5.4 kPa was reported from the first breath from a bottlenose dolphin after a 200 m dive, increasing to 11.9 kPa for the second breath (Ridgway et al., 1969). In the killer whale, the average O_2_ content was around 12-16 kPa for short apneas at all activity levels and decreased both with activity level and apnea duration, and the lowest measured levels were around 9.5 kPa (Figs 2-5 in B. Kriete, PhD thesis, The University of British Columbia, 1995). However, these values are not directly comparable to those collected in the current study or from Wahrenbrock et al. (1974) or Reed et al. (2000). In the deep diving dolphin and killer whale the air was collected in a bag and subsequently analysed for mixed average O_2_ for the entire breath. In the current study, on the other hand, the average expired O_2_ was the average of exhaled air during the exhalation. During the expiratory phase in bottlenose dolphins, respiratory flow increases rapidly to a more or less constant rate and O_2exp_ decreases until alveolar gas is sampled (Fahlman et al., 2015; Kooyman and Cornell, 1981; Mortola and Seguin, 2009). If we assume that the cumulative O_2_ taken up per breath is the integrated product of the instantaneous flow-rate and O_2exp_, we can estimate the expected average O_2_ content for each breath. In other words, the integrated volume of O_2_ taken up per breath and the measured VT can be used to more accurately to predict the expected average lung O_2_ content (Fig. 2B). This resulted in an average O_2_ content of all breaths of 15.2±1.8 kPa and a range from 9.2-19.8 kPa, which is similar to those of the killer whale (B. Kriete, PhD thesis, The University of British Columbia, 1995) and harbour porpoise (Reed et al., 2000), but higher than the values from the dolphin following a dive to 200 m (Ridgway et al., 1969). Thus, ΔO_2_ (10-11.6%) used in the previous studies (Armstrong and Siegfried, 1991; Blix and Folkow, 1995; Dolphin, 1987a; Folkow and Blix, 1992) is much higher than those observed in the dolphins. While Methods A and B may provide reasonable methods to estimate FMR, careful consideration of selected parameters are required and may explain the large error when compared with the dolphin during rest and recovering from exercise.

Using Eqn 1D allowed us to predict O_2exp_ during the recovery from exercise to account for the O_2_ debt that develops as the animal swims actively (Method C). This method allowed us to estimate the metabolic rate from breath numbers to within 12% following exercise. Diving mammals develop an O_2_ debt while foraging underwater as the internal O_2_ stores are used to fuel aerobic metabolism (Fahlman et al., 2008a; Kooyman and Ponganis, 1998; Scholander, 1940). Respiration rates often show a positive correlation with increasing activity (Blix and Folkow, 1995; Sumich, 1983) and dive depth and/or duration (Dolphin, 1987b; Würsig et al., 1986), and changes in exhaled gas content suggest that lung O_2exp_ decreases during the recovery (Boutilier et al., 2001; Fahlman et al., 2008a; Reed et al., 1994, 2000; Ridgway et al., 1969). Whether this relationship is similar following periods of prolonged apnea is likely but needs to be verified.

In summary, we used breath-by-breath respirometry to test if breathing frequency can be used to estimate metabolic rate in a smaller cetacean during rest and recovery from exercise. Our results suggest that the average O_2_ extraction and VT are lower than assumptions used in previous studies estimating FMR in mysticetes during migration and following diving (Armstrong and Siegfried, 1991; Blix and Folkow, 1995; Christiansen et al., 2014; Dolphin, 1987a,b; Folkow and Blix, 1992), contributing to overestimation by these particular methods. We found that accurate estimates of the average VT (Eqn 1B, Fig. 1B) and estimated O_2exp_ (Eqn 1D, Fig. 2B) content provided reasonable estimates of measured *V̇*_O_2__ (method C), to within 2% for pre-exercise rest and within 12% for post-exercise recovery data.

The two methods that used the upper limits for VT and O_2_ extraction overestimated measured oxygen consumption by 216-501%. One explanation may be the different behaviours investigated, where the previous studies focused on FMR during foraging, while in the current study we looked at the effect of rest and recovery from exercise. Individual breaths can be detected on acoustic bio-logging tags (e.g. Génin et al., 2015), and have been used to calculate breathing rates in free-ranging animals; however, the use of acoustic information (e.g. duration, frequency, or amplitude) from each breath has not been explored in its potential to provide measures of the variability critical for accurate estimates of energetics from breathing patterns (Sumich and May, 2009; van der Hoop et al., 2014b; our personal observations). Current work is underway to do so, and would greatly improve our ability to incorporate respiratory dynamics to properly estimate bioenergetics in free-ranging marine mammals. In addition, attempts are underway to determine if these relationships are valid following surface apneas and may shed additional information that may improve this potentially simple and powerful technique. Such physiological studies are crucial to determine the physiological dynamics, variability, and limitations of marine mammals and will enhance our ability to predict how they may respond to changes in the environment.

## MATERIAL AND METHODS

### Animals

All experiments were conducted under IACUC approval from the Animal Care Committee of Texas A&M University – Corpus Christi (AUP04-14), and the research committee of Dolphin Quest. Four adult male Atlantic bottlenose dolphins in a closed lagoon were used for all experiments; the name, body mass, straight length, and approximate age of the animals are summarized in Table 1. All experiments were performed using operant conditioning and participation by the dolphins was voluntary; animals were not restrained and could refuse to participate or withdraw at any point during experimental trials. Prior to initiating the study, animals were desensitized to the equipment and trained for novel research-associated behaviours. Thus, data on respiratory variables and metabolism were collected in dolphins that were in a relaxed, normal physiological state, before and after exercise.

### Respiratory flows

A custom-made Fleisch type pneumotachometer (Micah Brodsky, V.M.D. Consulting, Miami, FL) with a low-resistance laminar flow matrix (Item # Z9A887-2, Merriam Process Technologies, Cleveland, OH) was placed over the blowhole of the dolphin (Fig. 1 in Fahlman et al., 2015). Differential pressure across the flow matrix was measured using a differential pressure transducer (MPX-2.5 mbar type 339/2, Harvard Apparatus, Holliston, MA), connected to the pneumotachometer with two, 310 cm lengths of 2.0 mm I.D., firm walled, flexible tubing (see Figs 1 and 2 in Fahlman et al., 2015). The pneumotachometer was calibrated using a 7.0 litre calibration syringe (Series 4900, Hans-Rudolph Inc, Shawnee, KS). The signal was integrated and the flow determined by calibrating the pneumotachometer using the syringe immediately before and after each trial, through a series of pump cycles at various flow speeds (Fahlman et al., 2015) to calibrate the differential pressure and flows for the expiratory and inspiratory phases to be determined.

### Resting and post exercise metabolic rates

Each trial consisted of resting, exercise and recovery period. For the resting period, the dolphins rested (remained stationary) for at least 5 min, during which time the respiratory flows and expired gas composition were measured. The average O_2_ consumption rate (*V̇*_O_2__) was measured for the last 2 min of this resting period, during which time the animal was acclimated to the task and the *V̇*_O_2__ was stable and provided an accurate estimate of resting *V̇*_O_2__ (Fahlman et al., 2015). Next, the dolphin was instructed to either swim in a predetermined path for 10 min, or to swim behind a remote control model boat at a set pace of 3 m s^−1^ for 5 min. The dolphin was free to breathe at will while exercising. At the completion of the swimming task, the dolphin returned immediately to the measurement station, where post-exercise respiratory flow and expired gas composition were continuously measured for up to 5 min (see Fig. 3B in van der Hoop et al., 2014a).

### Respiratory gas composition

Respiratory gases were subsampled via a port in the pneumotachometer and passed through a 310 cm length of 2 mm I.D., firm walled, flexible tubing and a 30 cm length of 1.5 mm I.D. Nafion tubing, to fast-response O_2_ and CO_2_ analysers (ML206, Harvard Apparatus, Holliston, MA, USA) at a flow rate of 200 ml min^−1^, with a response time for a 90% change to equilibrium for O_2_ and CO_2_ of 67 ms and 94 ms, respectively (Fahlman et al., 2015). The gas analysers were connected to the data acquisition system and sampled at 200 Hz. The respiratory gas signals were phase-corrected to match the respirations, to account for the lag resulting from gas flow through the tubing. The expiratory flow-rate and expired O_2_ and CO_2_ content were multiplied to calculate the instantaneous *V̇*_O_2__ and carbon dioxide production rate (*V̇*_CO_2__, litre CO_2_ min^−1^). The instantaneous *V̇*_O_2__ and *V̇*_CO_2__ were integrated to yield the total volume of O_2_ and CO_2_ exchanged during each breath. The volumes were summed for each breath during the trial period and divided by the duration of the trial to provide an estimate of the *V̇*_O_2__ and *V̇*_CO_2__ for that time period.

The gas analyser was calibrated before and after the experiment using a commercial mixture of 5% O_2_, 5% CO_2_, and 90% N_2_, certified accurate to at least 0.01%. Ambient air was used to check the calibration before and after each experimental trial. Mean daily air temperature and humidity were 27.8±1.2°C (range 25.0–30.0°C) and 58.6±7.5% (46–77%). The average water temperature in the lagoon was 24.6±0.5°C. All gas volumes were converted to standard temperature pressure dry (STPD, Quanjer et al., 1993). Exhaled air was assumed saturated at 37°C, inhaled air volume was corrected for ambient temperature and relative humidity.

### Estimated *V̇*_O_2__ from breaths

Three different methods (A-C) were used to estimate *V̇*_O_2__ from the dolphins. Each model incorporated measured or assumed values to determine which variables have the greatest impact on the *V̇*_O_2__ estimate (Table 2). All methods (A through C) used the same basic equations to estimate *V̇*_O_2__, where the volume of O_2_ per breath (*V*_O_2__, litre O_2_) was estimated as:2A
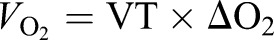
where VT is the tidal volume, and ΔO_2_ (% O_2_ breath^−1^) was determined as the difference in O_2_ concentration between inspired (O_2insp_, 20.94% O_2_) and expired (O_2exp_, %) air.2B
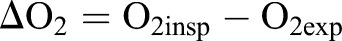
The total volume of O_2_ taken up per breath was calculated separately for the pre- and post-exercise periods, and divided by the total duration (t, min) of all breaths in that trial period to estimate the *V̇*_O_2__:2C
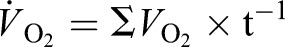
For method A, we used the assumption that VT is on average 80% of the vital capacity (VC), as estimated from Stahl's allometric equation (Stahl, 1967), and ΔO_2_ to be 10% (Armstrong and Siegfried, 1991; Dolphin, 1987a). For method B, we used the assumptions from those used to estimate FMR in minke whales (Blix and Folkow, 1995; Christiansen et al., 2014; Folkow and Blix, 1992), where VT was assumed to be 60% of the vital capacity, Eqns 4 and 7 in Folkow and Blix (1992), and ΔO_2_ was assumed to be 11.6%. For method C, we used the measured VT and integrated volume of O_2_ taken up during each breath to estimate the average O_2_ content for that breath, i.e. average O_2exp_2D
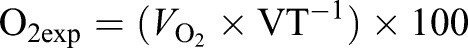
The estimated average O_2exp_ was used to determine average ΔO_2_, which, in turn was used to estimate *V*_O_2__ and *V̇*_O_2__ in method C.

### Data processing and statistical analysis

Metabolic data are reported as the average *V̇*_O_2__ for a trial. The relationship between a dependent variable and experimental covariates was analysed using mixed effects models (lme, R: A Language and Environment for Statistical Computing, R Foundation for Statistical Computing, version 3.1.0, 2014). Individual animals were treated as random effects to account for correlation between repeated measurements on the same individual (Littell et al., 1998). Best models were chosen by the Akaike information criterion (AIC) against the null model (AIC_null_) and significant parameters assessed by the t-value between the estimate and its standard error. *P*-values ≤0.05 were considered as significant. Data are presented as the mean±standard deviation (s.d.), unless otherwise stated.
